# Operationalising the Recovery College model with people living with dementia: a realist review

**DOI:** 10.1080/13607863.2024.2356878

**Published:** 2024-06-08

**Authors:** Melanie Handley, Charlotte Wheeler, Claire Duddy, Geoff Wong, Linda Birt, Chris Fox, Esme Moniz-Cook, Corinna Hackmann, Bonnie Teague, Juniper West

**Affiliations:** a Centre for Research in Public Health and Community Care, University of Hertfordshire, Hatfield, UK; b Research and Development, Norfolk and Suffolk NHS Foundation Trust, Norwich, UK; c Nuffield Department of Primary Care Health Sciences, University of Oxford, Oxford, UK; d School of Health Science University of East Anglia, Norwich, UK; e School of Healthcare, University of Leicester, Leicester, UK; f Medical School, University of Exeter, Exeter, UK; g Faculty of Health Sciences, University of Hull, Hull, UK; h Norwich Medical School, The University of East Anglia, Norwich, UK

**Keywords:** Dementia, Recovery Colleges, post-diagnostic support, co-production, realist review

## Abstract

**Objectives:**

Post-diagnostic support is a significant factor in facilitating personal recovery following a diagnosis of dementia, but access is often inconsistent and insufficient. Recovery Colleges offer peer-led, co-produced courses that can support people to have meaningful lives and have been adapted for use in the context of dementia. A realist review was conducted to understand the application and sustainability of Recovery College dementia courses.

**Method:**

An iterative, five-step process combined literature published to 2023 with knowledge from stakeholders with lived and professional experience of dementia involved with Recovery College dementia courses (PROSPERO registration CRD42021293687).

**Results:**

Thirty-five documents and discussions with 19 stakeholders were used to build the initial programme theory comprising of 24 context-mechanism-outcome configurations. Reoccurring factors included: attending to aspects of co-production and course delivery to ensure they promoted inclusion and were not compromised by organisational pressures; how stigma impacted access to course opportunities; and embedding personal recovery principles throughout course development to be relevant for people living with dementia and those who support them.

**Conclusion:**

People struggling to reconcile their future alongside dementia need practical and emotional support to access and benefit from Recovery College dementia courses, ways to achieve this will be explored through a realist evaluation.

## Introduction

Dementia is a condition characterised by a progressive decline in cognitive functions that impact a person’s everyday life. The onset of symptoms and subsequent diagnosis are life-changing, with people having to confront various material and emotional losses to make sense of what living with dementia means to themselves, their support network, and the future (Bunn et al., 2012; Hammond & Debney, 2017; Lee et al., 2014). Timely diagnosis of dementia is an international priority and a policy imperative in the United Kingdom (UK) (Alzheimer’s Society, 2023; Department of Health, 2008; WHO, 2017).

In the UK, diagnosis often takes place in Memory Clinics that are usually set within National Health Service (NHS) mental health services and run alongside Community Mental Health Teams for Older People. For many people and families, this may be their first contact with mental health services (Manthorpe et al., 2018). Memory Clinics were established as a means of reducing the fear and stigma associated with ageing, mental health, and dementia (Moniz-Cook & Mountain, 2023). However, the negative effects of stigma, co-located memory and mental health services, and the view that there is ‘nowhere to turn to’ appear to be ongoing barriers to accessing support opportunities for people and their families (Giebel et al., 2021; Moniz-Cook & Mountain, 2023; Stephan et al., 2018). Many people report being left to make sense of the condition without professional support (Birt et al., 2023; Horik et al., 2022). Access to services that can assist a person to assimilate dementia as part of their identity, minimise the impact of cognitive decline, and neutralise the effects of stigma, shame, or other negative internalised concepts (Herrmann et al., 2018) are urgently needed. One emerging model of post-diagnostic support that aims to help people understand dementia and learn ways to live as well as possible with the condition is Recovery Colleges. The first UK Recovery College was established in 2009. The model has since been adopted by over 80 organisations; predominantly NHS mental health services and some third sector providers (Hayes et al., 2023; Wolverson et al., 2023). The Recovery College model represents a core but different, complementary approach to clinical mental health services, and most Colleges for adults with mental health conditions run courses from various community locations, or online, using a ‘hub and spoke’ approach from a central base (Perkins et al., 2017).

Recovery Colleges offer educational courses that are co-designed and co-led—’co-produced’—with people who have a lived experience of mental health difficulties, known as peer tutors, and healthcare staff. Courses aim to support people in their personal rather than clinical recovery by building their knowledge and confidence to self-manage their conditions and live meaningful lives. Based on the principles of inclusion and equity, courses are co-produced to address the five domains of the CHIME framework of personal recovery: Connectedness, Hope, Identity, Meaning, and Empowerment (Leamy et al., 2011). Evidence related to the value of Recovery Colleges for both service users and organisations is growing (Thériault et al., 2020) and the model is being adapted for different populations, including for people living with dementia (Wolverson et al., 2023).

Recovery College courses about dementia are a recent phenomenon and as such, there is limited understanding for how the values of Recovery Colleges, i.e. those of co-production, peer tutoring, and the notion of personal recovery, can be applied to the experience of a progressive condition, such as dementia. Co-production of educational courses, with opportunities to connect and learn directly with peers, are core to the ethos of the Recovery College model (Perkins et al., 2012). Meaningful involvement and ownership of these activities from people living with dementia are likely to be established when factors related to a person’s cognitive, physical, and psychosocial needs are attended to throughout course development. Person-centred approaches (Brooker & Latham, 2015; Kitwood, 1997) are recommended when working with people with dementia and may be useful guiding principles for Recovery College dementia courses. This review aimed to build an initial programme theory to understand how Recovery College dementia courses are developed, used, and sustained within publicly-funded mental health services. This is warranted given increasing demands for affordable post-diagnostic support that can help people maintain meaningful lives alongside dementia.

## Methods

The multiple, interconnected factors of psychosocial interventions, such as Recovery College dementia courses, can be expected to result in a range of outcomes across stakeholders and settings. Therefore, this requires a research methodology that can incorporate complexity rather than control for it. Realist review is a theory-driven approach to evidence synthesis that seeks understanding beyond whether or not an intervention works, to build and refine programme theories of what works, for whom and in what circumstances (Pawson, 2006). Realist review can be used to investigate novel interventions where the evidence-base is limited but evolving. Through systematic and iterative engagement with diverse sources, realist review builds explanations from relevant evidence across conceptually linked interventions and existing theories. The understanding captured in the programme theory can inform subsequent research and recommendations for intervention design, policy, and practice.

**Figure 1.**
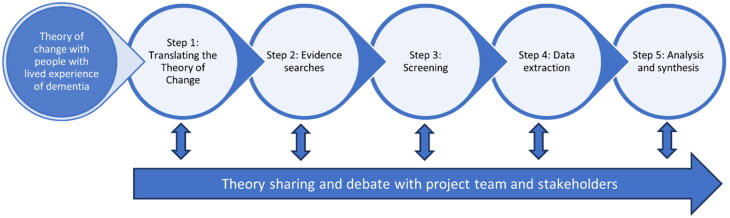
The realist review process.

Realist review was used to develop an initial programme theory of Recovery College dementia courses that would inform later stages of the DiSCOVERY project (NIHR131676, 2022–2024). An iterative five-step process, adapted from Pawson’s suggested steps (Pawson, 2006), drew on published evidence, knowledge from the project team, and consultation with stakeholders with lived and professional experience of dementia and Recovery Colleges (Figure 1).

To ensure timely completion, certain review processes were truncated. Specifically, in Step 2 evidence searches were highly focused and in Step 3 the eligibility criteria were tighter. The protocol was published on PROSPERO (CRD42021293687) and follows the Realist And Meta-narrative Evidence Syntheses: Evolving Standards (RAMESES) quality and publication standards (Wong et al., 2014).

### Step1: Translating an existing theory of change

In work preceding the current study, a group comprised of people living with dementia, their family supporters, and psychologists working in memory services collaboratively developed and ran a Recovery College dementia course. As part of creating the course, the group co-produced a theory of change (Supplementary Materials 1 and 2). The theory of change was translated into a set of propositions by one reviewer (MH), debated with the project team in two online meetings, and updated accordingly.

### Step 2: Evidence searches

Literature searches focused on three categories:

1. Recovery College dementia courses.
2. Recovery Colleges in general.
3. Recovery in dementia.

An information specialist with expertise in realist review (CD) designed and conducted searches of four electronic databases (MEDLINE, Embase, PsychINFO, CINAHL), Google Scholar, and a range of relevant websites (Dementia Engagement and Empowerment Project (DEEP), Implementing Recovery through Organisational Change (ImROC), NHS Evidence, NIHR Library) (Supplementary Material 3). To supplement the limited data, additional searches informed by Step 1 were used to identify material that described tutor and student experiences of Recovery Colleges in other contexts.

### Step 3: Screening

**Figure 2.**
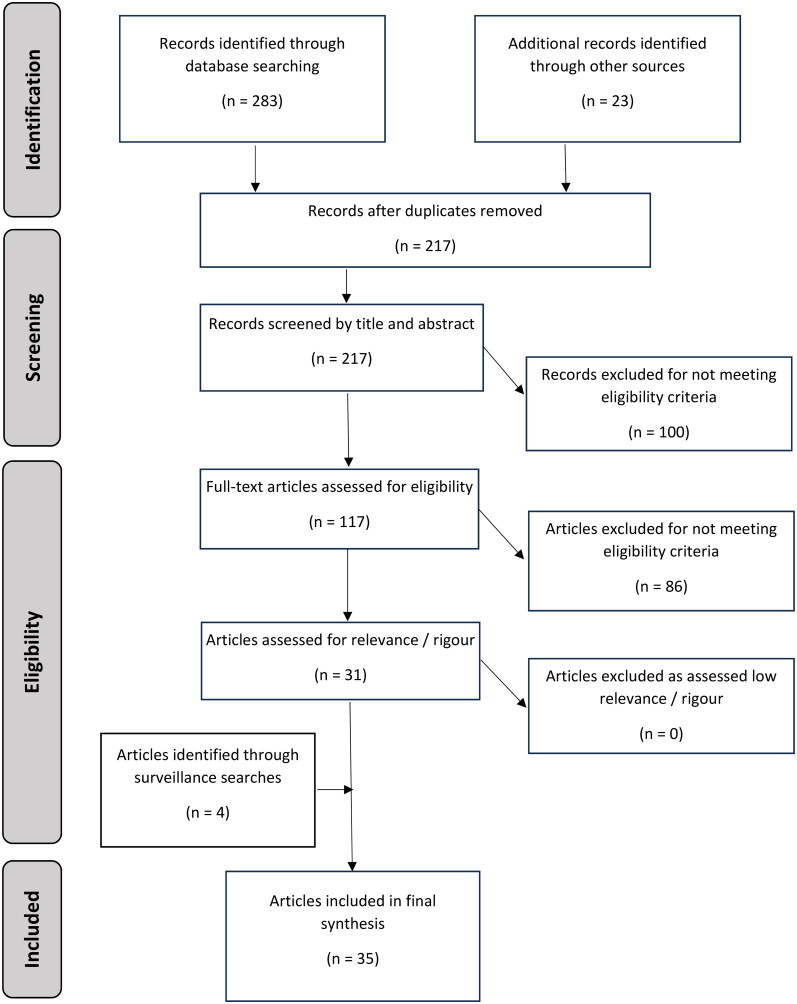
PRISMA flow diagram of searches and evidence retrieval.

Figure 2 sets out the selection process. Results were exported to reference management software (Endnote X9) and duplicates were removed. Results were screened by one reviewer (CW) with a 10% random subsample screened in duplicate by a second reviewer (JW) to ensure consistent application of inclusion and exclusion criteria.

For the core category of Recovery College dementia courses, inclusion criteria were:

- Documents describing the implementation, outcomes, or experience of Recovery Colleges or other ­co-produced post-diagnostic support for dementia within mental health services;
- No limits were placed on study design, document type, or date of publication.

For the category of Recovery College courses with other populations, inclusion criteria were:

- Papers describing the experience of co-producing and using Recovery Colleges;
- Evidence related to theory building, for example, Recovery College characteristics and course outcomes;
- Study designs of interest were qualitative, mixed methods, and evaluations.

Inclusion category for recovery in dementia were:

- Evidence related to how recovery approaches were applied with people living with dementia.

The Recovery College model has distinctive features that influence how the co-production of courses occur with people with lived and professional experience. While realist reviews may include conceptually linked evidence, the aim is to build credible programme theories with the potential for practical application within a system of interest, in this case, mental health systems. Therefore, we excluded literature reporting general principles of co-production or co-production with people living with dementia undertaken outside mental health settings.

Full texts were screened by a single reviewer (CW), with support from a second reviewer (MH). Realist review criteria of relevance and rigour were applied for selecting and appraising documents. Decisions were recorded in an Excel sheet.

### Step 4: Data extraction

A single reviewer (CW) extracted data from documents as follows:

- descriptive data describing the included documents (e.g. date, type of document, study design) were tabulated in Excel
- full-text documents were uploaded into qualitative data analysis software (NVivo) and coded both deductively, using propositions developed in Step 1, and inductively.

Another research team member (MH) checked a 10% random sample for systematic errors. Inconsistencies were discussed and resolved in meetings with the core review team (CW, MH, CD, GW).

### Step 5: Analysis and synthesis

A single reviewer (MH) led data analysis and synthesis with regular discussions with the research team to challenge and debate interpretations. Realist logic of analysis was applied to develop propositions into context-mechanism-outcome configurations (CMOCs) across the development and delivery pathway of Recovery College dementia courses for ­co-producers and attendees with lived and professional ­experience of dementia.

### Stakeholders

Four online meetings were convened with stakeholders with experience of co-producing or attending Recovery College dementia courses. Meetings were organised to separately host stakeholders who represented:

- people with lived experience of dementia
- people working in services used by people with dementia.

People living with dementia were recruited through the Dementia Engagement and Empowerment Project (DEEP, https://www.dementiavoices.org.uk/) and include representatives from across the United Kingdom.

During meetings, the developing theories were explored. These discussions were recorded, transcribed verbatim and relevant data were analysed to further refine the initial programme theory. Ethical approval for the collection of stakeholder data was obtained from HRA and Health and Care Research Wales (ref 22/WM/0021). Each attendee gave consent.

## Results

### Description of evidence

Evidence from 35 papers and blog posts (Table 1) was supplemented by discussions with 19 stakeholders involved with Recovery College dementia courses (ten people with lived experience and nine staff) across four meetings to develop a series of CMOCs that set out the initial programme theory. Documents were published between 2012 and 2023, with two-thirds (*n* = 23) published since 2018. Included documents were peer-reviewed journal articles (*n* = 24), reports (*n* = 3), briefing paper (*n* = 1), blog posts (*n* = 5), doctoral thesis (*n* = 1), and conference presentation (*n* = 1). The majority of documents (*n* = 32) reported experiences, characteristics, or guidance for Recovery Colleges, although only eight of these, which included four blog posts, focused on Recovery College dementia courses. Two documents discussed co-production of services and groups with people living with dementia and one document explored the use of recovery approaches for older people including those living with dementia. Thirty-two documents were from the UK, one from Italy, one from Canada and one document included data on Recovery Colleges across multiple countries. All documents contributed to at least one CMOC, with stakeholder discussions enhancing understanding for 20 CMOCs (14 from stakeholders with lived experience of dementia and 19 from staff stakeholders).

**Table 1.** Evidence contributing to the initial programme theory.

No	Author	Year	Title	Document category	Country	Type of article	Method	Included in CMOC	Participant type
1	Ali, Imran, et al.*	2022	Reflections on co-production, lived experience and the shared learning environment within the development and early delivery of a Recovery College	Recovery College General	UK	Peer-reviewed article	Qualitative	10, 20, 22	4 Service users 4 Service users and staff 2 Carers 2 Volunteers 13 Staff Total 25
2	Burhouse et al.	2015	Coaching for recovery: a quality improvement project in mental healthcare	Recovery College General	UK	Peer-reviewed article	Qualitative	6, 7, 19, 20	Not reported
3	Cameron et al.	2018	Collaboration in the design and delivery of a mental health recovery college course: experiences of students and tutors	Recovery College General	UK	Peer-reviewed article	Case study	2, 3, 7, 9, 11, 19, 22, 24	2 Service user tutors 2 Staff tutor 9 Service user students (Additional naturally occurring data: 3 tutors and 45 students)
4	Cheffey et al.	2017	“Can I facilitate a project when my memory lets me down?”: The challenges and rewards of co-producing a “living well with dementia” course	Recovery College dementia course	UK	Report	Descriptive account	5, 7, 8, 10, 11, 12, 20, 21, 22	1 Lived experience tutor 1 Staff tutor
5	Crowther et al.	2019	The impact of Recovery Colleges on mental health staff, services and society	Recovery College General	UK	Peer-reviewed article	Realist review	1, 7, 12	N/A
6	Dalgarno and Oates	2019	The crucible of co-production: Case study interviews with Recovery College practitioner trainers	Recovery College General	UK	Peer-reviewed article	Case study	9, 10, 12, 23	8 Staff tutors
7	Dalgarno and Oates	2018	The meaning of co-production for clinicians: An exploratory case study of Practitioner Trainers in one Recovery College	Recovery College General	UK	Peer-reviewed article	Qualitative interviews	3, 8, 12	8 Staff tutors
8	Daley et al.	2013	Development of a framework for recovery in older people with mental disorder	Recovery and dementia	UK	Peer-reviewed article	Qualitative interviews	6, 22	38 Service users
9	Duff	2016	Exploring the use of a recovery college for older people with dementia in the UK	Recovery College dementia course	UK	Conference presentation	Descriptive account	2, 3, 22	N/A
10	Hayes et al.*	2022	Recovery Colleges Characterisation and Testing in England (RECOLLECT): rationale and protocol	Recovery College General	UK	Peer-reviewed article	Protocol	12	N/A
11	Hayes et al.*	2023	Evidence‑based Recovery Colleges: developing a typology based on organisational characteristics, fidelity and funding	Recovery College General	UK	Peer-reviewed article	Survey	2, 19	88 Recovery Colleges
12	Kenny et al.	2016	Facilitating an evolving service user involvement group for people with dementia: what can we learn?	People with dementia involvement in service design	UK	Peer-review article	Case study	1, 6	5 Service users with dementia 4 Staff 2 Students
13	Lowen et al.	2019	Recovery Colleges and dementia courses–A scoping survey.	Recovery College dementia courses	UK	Peer-review article	Scoping review	2, 10, 13	N/A
14	Lucchi et al.	2018	Programma FOR: a recovery college in Italy.	Recovery College General	Italy	Peer-reviewed article	Mixed methods evaluation	2	34 Service users attendees 20 Staff tutors
15	McGregor et al.	2016	Recovery Colleges International Community of Practice Proceedings of June 2015 Meeting. RCICoP Group.	Recovery College General	Multiple	Report	Consensus meeting	7, 8, 11, 12, 20	Not reported
16	Meddings et al.	2019	To what extent does Sussex Recovery College reflect its community? An equalities and diversity audit.	Recovery College general	UK	Peer review article	Service evaluation	18, 19	N/A
17	Meddings et al.	2014	Co-delivered and co-produced: creating a recovery college in partnership	Recovery College General	UK	Peer-reviewed article	Action research	1, 4, 7, 8, 9, 23	Not reported
18	Perkins, AM et al.	2017	Impacts of attending recovery colleges on NHS staff	Recovery College General	UK	Peer-reviewed article	Survey	23	100 Staff attendees
19	Perkins, R et al.	2012	Briefing paper: Implementing Recovery through Organisational Change	Recovery College General	UK	Briefing paper	Defining Recovery Colleges	5, 7, 13	N/A
20	Perkins, R et al.	2017	Recovery colleges 10 years on	Recovery College General	UK	Report	–	1, 2, 19	N/A
21	Reid et al.	2020	Mechanisms of change and participant outcomes in a Recovery Education Centre for individuals transitioning from homelessness: a qualitative evaluation	Recovery College General	Canada	Peer-reviewed article	Realist evaluation	20, 22	20 Course attendees
22	Skipper and Page	2015	Our recovery journey: two stories of change within Norfolk and Suffolk NHS Foundation Trust.	Recovery College General	UK	Peer-reviewed article	Narrative account	1, 4, 5, 9	1 Staff tutor 1 Service user tutor
23	Sommer et al.	2018	Walking side-by-side: Recovery Colleges revolutionising mental health care	Recovery College General	UK	Peer-reviewed article	Qualitative focus groups	1, 23	16 Service users 13 Staff
24	Thompson et al.	2021	Recovery colleges: long-term impact and mechanisms of change	Recovery College General	UK	Peer-reviewed article	Qualitative interviews	20	14 Course attendees
25	West et al.	2022	A case study of co-production within a mental health Recovery College dementia course: perspectives of a person with dementia, their family supporter and mental health staff	Recovery College dementia course	UK	Peer-reviewed article	Case study	1, 3, 5, 6, 7, 11, 12, 19	2 Staff tutor 2 Lived experience tutor (1 living with dementia, 1 family carer)
26	Whish	2019	How do clinicians experience their role in recovery colleges?	Recovery College General	UK	Unpublished Thesis	Qualitative interviews	3, 9, 11	11 Recovery College Staff
27	Wolverson et al.*	2023	Building an initial understanding of UK Recovery College dementia courses: a national survey of Recovery College and memory services staff	Recovery College dementia course	UK	Peer-reviewed article	Survey	2, 18	261 Staff
28	Yoeli et al.	2022	Recovery in Mind: A Recovery College’s journey through the Covid-19 pandemic	Recovery College General	UK	Peer-reviewed article	Mixed methods	1, 2, 5	89 Students
29	Zabel et al.	2016	Exploring the impact of the recovery academy: a qualitative study of Recovery College experiences.	Recovery College General	UK	Peer-reviewed article	Qualitative interviews	1, 15, 18, 19, 20	9 Service user attendees 1 Family carer attendee 14 Staff attendee
30	Zucchelli and Skinner	2013	Central and North West London NHS Foundation Trust’s (CNWL) Recovery College: the story so far….	Recovery College General	UK	Peer-reviewed article	Narrative account	1, 2, 5, 7, 11, 12	N/A
31	George, Dementia Diaries	2021	What are the qualities of an effective DEEP group facilitator? George muses on this as he steps outside again.	Co-production with people living with dementia	UK	Blog post		8, 20	N/A
32	Wendy Mitchell	2018	Which me am I today? Three more wonderful opportunities…	Recovery College dementia course	UK	Blog post		16	N/A
33	Wendy Mitchell	2021	Which me am I today? Recovery College time	Recovery College dementia course	UK	Blog post		16	N/A
34	Wendy Mitchell	2021	Which me am I today? Living with dementia and things you can do to help	Recovery College dementia course	UK	Blog post		20	N/A
35	Wendy Mitchell	2020	Which me am I today? New induction, new format	Recovery College dementia course	UK	Blog post		23	N/A
	Stakeholder discussions: People with lived experience of dementia and Recovery Colleges							6, 7, 8, 12, 13, 14, 15, 16, 17, 20, 19, 20, 21, 22	10 people with lived experience of dementia and co-producing Recovery College dementia courses
	Stakeholder discussions: staff							3, 4, 5, 6, 7, 8, 10, 11, 12, 13, 15, 16, 17, 18, 19, 20, 21, 22, 24	9 staff

* Papers added through surveillance searches.

### Initial programme theory for co-producing, co-facilitating, and sustaining Recovery College dementia courses

**Figure 3.**
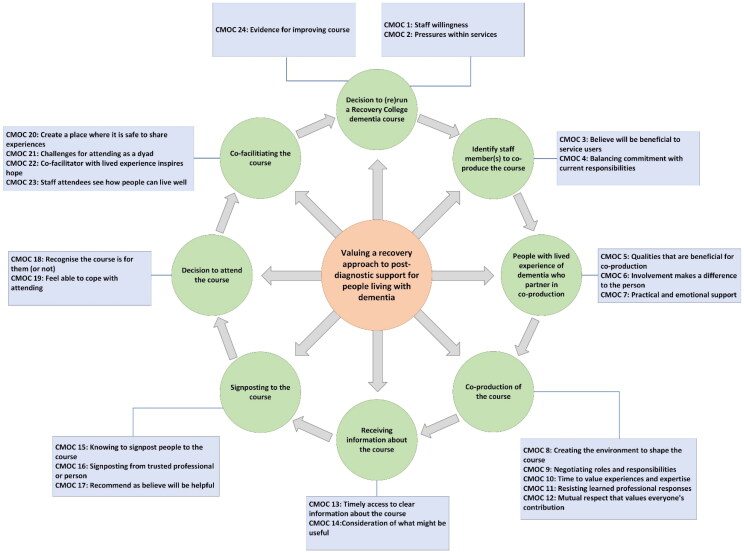
Initial programme theory of co-producing Recovery College dementia courses.

Nine steps related to 24 CMOCs set out the factors linked to co-producing and co-facilitating a Recovery College dementia course (Figure 3 and Table 2). CMOCs 1–4, 23, and 24 set out explanations common to other Recovery College courses but are limited in their specificity to dementia courses so have not been included in the explanation below. However, these CMOCs have been included in Figure 3 and Table 2 as their relevance to Recovery College dementia courses will be explored in the realist evaluation (Birt et al., 2023). The narrative account below focuses on CMOCs 5–22 to present how the interlinked explanatory CMOCs influence course outcomes and for whom.

**Table 2.** Context-mechanism-outcome configurations of the initial programme theory.

Decisions to run a Recovery College dementia course	
CMOC1	When services see the benefit of Recovery College principles for mental health conditions (C), they are willing to consider the model for people living with dementia (O) because they recognise its value (M).
CMOC2	Financial instability and staffing constraints (C) means services are less likely to allocate resource to co-produce courses (O) because there are more pressing priorities (M).
Identify staff member(s) to co-produce the course	
CMOC3	Staff who value Recovery College principles (C), are more likely to initiate co-production of courses (O) because they believe it will be helpful for people they work with (M).
CMOC4	Staff willing to co-produce courses and have support to undertake the role (C) will be able to balance the commitment alongside their day-to-day work (O) because they feel able to incorporate the additional work in their role (M).
People with lived experience who partner in co-production	
CMOC5	When staff working with a person in a clinical capacity consider that co-producing a course will benefit their personal recovery (C) the person will be invited co-produce the course (O) because they believe they have the necessary attributes to cope with the demands (M).
CMOC6	When peer tutors see that sharing their experience of coping with dementia is useful to other people (C) they feel better about themselves and have a sense of purpose (M) that helps restore their sense of identity (O).
CMOC7	Practical and emotional support (C) will mean people with dementia directly shape and deliver course content (O) because they feel more confident to do so (M).
Co-production of the course	
CMOC8	Co-producers who spend time discovering how to work together (C) can overcome traditional professional/client boundaries and create an environment for honest conversations that shape the course (O) as people feel listened to, respected and supported (M).
CMOC9	Co-producers with existing relationships from clinical work (C) may initially find it difficult to work together on the course (O) as they may feel vulnerable negotiating the change to their roles, responsibilities and identities (M).
CMOC10	When staff have enough time for course co-production (C), they will share the responsibilities and ownership of course development with peer tutors (O) because there is space to value each person’s experiences and expertise (M).
CMOC11	Staff who can adapt clinical skills to co-production (C) will create a space conducive to course development (O) because they resist learned professional urges and instead reflect on the skills and approach is needed (M).
CMOC12	Staff with mutual respect for the expertise of co-producers (C) will inform and improve their clinical practice (O) because they are open to hearing different perspectives (M).
Receiving information about the course	
CMOC13	If information about a course is available at a time and in a format that suits a person’s needs (C) they can make an informed decision for attending (O) because they will feel able to assess the benefit for themselves (M).
CMOC14	Receiving information about a course at the same time as being diagnosed with dementia (C) is unlikely to encourage attendance (O) because people feel overwhelmed with information (M) and need time to consider if the course will be useful to them (M).
Signposting to the course	
CMOC15	If partnership organisations and local professionals know about a course and discuss the opportunity with people with dementia they work with (C) this will raise awareness of the course through an established relationship (M) making it more likely a person will attend (O).
CMOC16	When people hear about the potential benefits of a from someone they trust (C) they are more likely to attend (O) because they value and believe the source (M).
CMOC17	Professionals who understand the benefits of the course (C) are more likely to recommend them (O) because they believe it will be helpful (M).
Decision to attend the course	
CMOC18	Misunderstanding the purpose of Recovery College dementia courses (C) will reduce attendance (O) because people do not see the course as relevant (M).
CMOC19	By ensuring the length, format and venue of a course are suitable for the intended audience (C) the course will more likely appeal (O) as people feel able to cope with attending (M).
Co-facilitating the course	
CMOC20	Skilled co-facilitators who create a place where contributions are listened to and respected (C) will enable people to learn from each other (O) because they feel safe to share their respective experiences (M).
CMOC21	When people attend as a dyad (person with dementia and their supporter) (C) it may be difficult for them to be honest about their experiences (O) because they want to protect the other person and not upset them (M).
CMOC22	When a peer tutor with dementia is able to show others how they live with dementia (C) people with dementia may being to recover a sense of control over their lives (O) because they learn to reconcile their view of themselves and how to live with their diagnosis (M).
CMOC23	Staff who attend a course co-facilitated by a person with dementia (C) will see what it means to live well with dementia and consider more holistic ways to support people (O) because they come to appreciate what people with dementia are capable of (M).
Decision to rerun a Recovery College dementia course	
CMOC24	Course evaluation (C) can inform changes needed to reach those who will benefit and improve course content (O) because staff have the relevant data (M).

#### People with lived experience of dementia who partner in co-production

Existing clinical relationships between mental health staff co-producing Recovery College dementia courses and people living with dementia were used to recruit co-producers with lived experience (Cheffey et al., 2013; West et al., 2022). Staff approached people they considered would benefit from work that moved beyond the current therapeutic relationship to one where the person actively applies their skills, experience, and knowledge to co-producing a course (CMOC5). In this way, people with lived experience who co-produced Recovery College courses recognised how their contributions were useful, which gave them a sense of purpose and helped them to come to terms with their own diagnosis (CMOC6).

Staff identified several core characteristics of the people living with dementia invited to co-produce courses. Besides having experiences of dementia that other people could relate to, the person’s ability to articulate their experiences of coming to terms with their dementia diagnosis was important. Additionally, staff needed to believe the person would be able to cope with sharing their story in a mixed group setting (Cheffey et al., 2013). Recovery College dementia courses are attended by people with dementia, people supporting someone with dementia, health and social care staff, and in some cases, the public. Sharing difficulties might make a person feel vulnerable and not everyone attending courses would necessarily identify with someone else’s experience or react with compassion towards them. Furthermore, there could be conflicting viewpoints from people attending the course. Stakeholders discussed some of the practical and emotional support that staff could provide to prepare peer tutors to co-facilitate the course (CMOC7). This included agreeing responsibilities for delivering course content and facilitating the group, and knowing each other well enough to be able to intervene when necessary.

#### Co-production of the course

ImROC values and principles of practice for co-producing Recovery College courses emphasise the importance of equitable relationships between staff and peer tutors to challenge the traditional power dynamic between clinicians and service users (Perkins et al., 2012). In clinical consultations, staff are usually positioned as the expert, devising care and treatment plans often for, rather than with, service users. For ­co-production to be meaningful, mutual recognition of the qualities, expertise, and skills of all co-producers contributing to the development of the course was important. It could take time to achieve new ways of working (CMOC8), particularly where staff and service users had an existing professional relationship (CMOC9). People spoke of the challenges in negotiating these new relationships. For staff, this could be unsettling as they relinquished the power that came with their clinical role to share course creation and decision making with the group (Whish, 2021). For people with lived experience, the challenge was to be recognised as an equal partner with valuable knowledge that would contribute to developing the course.

Staff stakeholders acknowledged that given the restricted amount of time they had for co-producing the course along with deadlines for presenting course proposals to colleagues, that intentions for greater levels of co-production could be difficult to maintain (CMOC10 and 11). However, stakeholders with dementia felt strongly that co-production should not be reduced to tokenistic displays of involvement. Agreeing how people would work together, each person’s responsibilities for developing the course and a realistic understanding of the degree of co-production possible were considered important (CMOC12). In this way, people’s expectations were managed while helping each person feel ownership of the process and commit to deliver the course.

#### Reaching people who will benefit from the course

Post-diagnostic support in the form of Recovery College dementia courses aimed to help people live as well as possible with their condition. However, stakeholders reported that feeling emotionally able to attend and benefit from the course was a major challenge. Therefore, information needs went beyond practicalities related to course detail (CMOC13). Providing information about a course at the point of diagnosis was considered important but unlikely to be a time that someone would be able to contemplate attending a course (CMOC14). The experience of receiving a diagnosis of dementia was discussed by stakeholders as overwhelming and devastating. This was accompanied by an excessive amount of information imparted at the time of diagnosis, probable discharge from memory services, and no planned follow-up. The sense of being abandoned to cope with the diagnosis alone and internalised stigma contributed to feelings of shame and hopelessness that were barriers to seeking help.

Additional avenues for sharing information were considered necessary not only to raise awareness of the course but also to discuss people’s concerns about attending and to provide reassurances that the course had been designed for them. Therefore, it was considered important that Recovery Colleges worked with a network of professionals from primary and secondary care, local authority, and third sector organisations working with people with dementia to promote the course (CMOC15). Stakeholders reported that learning about a course from someone who’s opinion they trusted helped overcome their concerns which led them to register for a course (CMOC16). However, whether a professional shared information about a course depended on their perception of the benefit for the person and the professional’s own understanding of Recovery Colleges (CMOC17).

#### Decision to attend the course

Several debates in the literature, with stakeholders, and from blogs related to the use of recovery and educational language used to advertise course material. While the principles of personal recovery for dementia were understood by those involved in co-producing Recovery College courses (that is recovering a meaningful and fulfilling life following diagnosis), there were concerns that others would misinterpret the concept or that it could be off putting (CMOC18). Currently, Recovery College dementia courses advertise courses using titles, such as ‘Living well with Dementia’ (Wolverson et al., 2023), although people’s interpretations of ‘Living well’ are subjective and dependent on their personal circumstances and therefore may raise similar concerns (Clare et al., 2019).

The general Recovery College literature described how the course venue, format, and associated materials could facilitate learning opportunities and help challenge traditional assumptions about expertise and knowledge. Stakeholders highlighted that insufficient consideration of these practical factors could exclude people from or during the course. Both the environment and the organisation of the course needed to accommodate cognitive challenges related to dementia as well as other likely co-occurring needs linked to mobility, sensory issues, or other health conditions (CMOC19).

#### Co-facilitating the course

Co-facilitators of Recovery College courses needed to create an emotionally safe environment where attendees felt able to respond honestly to the course content and share their own experiences with the group (CMOC20). This was considered important for people to fully benefit from attending the course and learn from each other. For dementia courses, co-facilitators needed to carefully consider how to manage a group comprising of people with dementia, family/friend supporters, and staff. Literature and stakeholders raised how people attending in these different capacities might share experiences or opinions that are challenging for others in the group. For people attending as a dyad of a person with dementia and their supporter, there may also be a reluctance to speak about situations for fear of offending and upsetting the person they are attending with (CMOC21). As with other courses, skilled co-facilitation could enable inclusion by providing opportunities for everyone to contribute if they felt comfortable to do so. Important strategies for attendees with dementia were noticing signs that people want to say something and asking them about it directly, reducing the time people had to wait to share their thoughts during group discussions, and managing the dynamics with their supporter who may be used to speaking on the person’s behalf. These approaches could help people with dementia feel listened to and able to actively participate in the course.

Course content related to living with dementia and delivered by someone personally affected by the condition was thought to strengthen learning and discussion among attendees. A peer tutor described connecting with attendees with dementia through their shared experiences of living with the condition. Stakeholders believed people with dementia would benefit from attending courses that facilitated exploration of what it means for them personally to live with the condition. In this way, individuals could ‘recover’ a sense of control for how they managed their lives. By seeing someone with dementia co-facilitating a course, attendees with dementia were able to consider the possibilities and their future more positively (CMOC22).

## Discussion

Recovery College dementia courses are an opportunity to expand, the currently somewhat limited, post-diagnostic provision by focusing on how people can adapt to living with dementia. This realist review sets out an initial programme theory for co-producing courses and factors related to their sustainability. While there was limited published evidence specific to Recovery College dementia courses, the wider Recovery College literature, debates around recovery in the context of dementia, and discussions with stakeholders enabled us to articulate what works, for whom, and in what circumstances across the pathway of course development and delivery. Several factors recurred across these stages, with likely impacts on who benefits from Recovery College dementia courses and in what way. These included: ensuring that methods and materials for co-producing and co-facilitating the course were inclusive of different people’s needs, the applicability of recovery approaches with people living with dementia, and how stigma could affect access to co-production opportunities and attending courses.

The sustainability of Recovery College courses and similar peer-led initiatives in the community is a challenge (Morton et al., 2021; Wolverson et al., 2023). Financial and staffing resource constraints on secondary mental health services, the lack of specific commissioning of colleges by the NHS and the enduring consequences of COVID-19 mean post-diagnostic interventions are vulnerable to not being seen as core provision (Hayes et al., 2023). Further issues of sustainability for Recovery College dementia courses relate to the continued involvement from people who live with progressive conditions and how changes to a person’s circumstances can be managed sensitively within post-diagnostic support service design. There was limited discussion of these aspects of sustainability in the literature, likely due to the recent adoption of the model in the context of dementia. Most evidence related to the motivations of co-producers with lived experience of dementia becoming and continuing to be involved with courses; learning which could assist the recruitment of co-producers. Further understanding is needed to inform guidance on how to support people as their ability to cope with the demands of course development and delivery changes.

Co-production is a core characteristic of Recovery College courses (Toney et al., 2019). There was limited description in the literature of how co-production is negotiated and facilitated between staff and people with lived experience of dementia within the constraints of the Recovery College model. Most evidence was generated with stakeholders with experience of co-producing courses. These discussions and evidence from wider Recovery College literature explored the tensions for achieving co-produced courses associated with individual and service level factors. Practical considerations for ensuring equity in co-production, such as timing, venue for meetings, and materials that aided the continuity of discussions, were easier to identify and define. Supporting a person’s psychosocial needs was subtler, often relying on the quality of the relationship between staff and lived experience tutors, which may not be linear and may be vulnerable to the pressures of developing the course. In these circumstances, and similar to co-production in other areas of health care (Bosco et al., 2019), values for equality in the partnership may suffer. Understanding how to adapt course co-production for a population that is likely to be older than other Recovery College populations and be living with cognitive and functional decline alongside other health conditions is needed.

The literature and conversations with stakeholders debated the tension of using the language of recovery in the context of dementia. Those familiar with the work of Recovery Colleges, and more broadly recovery-focused practice (e.g. Woods, 2007), felt the link was appropriate. However, they recognised the difficulty arose from how the concept ‘recovery’ could be interpreted. The conventional clinical and lay definition relates to the cure of a disorder and/or return to previous levels of functioning, whereas personal recovery involves changes in attitudes, hope, values, and goals to live, where possible, a meaningful and fulfilling life (Hammond & Debney, 2017). Where people assumed the first definition, there were concerns this could lead to unrealistic expectations for what a Recovery College dementia course could achieve. To navigate this, Recovery College dementia courses have avoided recovery terminology in their course materials, substituting these with phrases, such as ‘living well with dementia’. The terms used are potentially less important than how the principles of recovery, as defined through the CHIME framework, inform the course (Hill et al., 2010). For Recovery College dementia courses, this relates to understanding how co-production, inclusion, and strength-based approaches can assist people to (re)establish social connection, optimism about the future, meaning and purpose in life, agency and empowerment, and critically, assimilate the diagnosis into a sense of self or identity.

### Implications for policy, practice, and future research

A key factor affecting if and when people access support through Recovery Colleges was stigma; this was mainly discussed by stakeholders living with dementia. Experiences of shame and stigma have been associated with the avoidance of participating in activities and accessing health services (Aldridge et al., 2019; Ryan, 2021). These experiences may be compounded by attitudes, beliefs, and perceptions related to mental health difficulties and further impact course attendance. Previous studies highlight that many people with dementia are uncomfortable attending mental health settings, preferring more familiar and less stigmatised locations (Stephan et al., 2018). This has implications for policy and practice for post-diagnostic services aiming to help people with these experiences when attendance is voluntary and dependent on a person seeking help, rather than facilitated as part of care planning discussions following diagnosis. Methods for raising awareness of Recovery College dementia courses and encouraging people to attend will likely need to be multifaceted, with the relevance and benefits of the course to the person reinforced by trusted professionals and peers. Understanding how best to encourage people living with dementia experiencing stigma and shame to access ­post-diagnostic support is an important aspect of future research and will be explored during the realist evaluation component of DiSCOVERY (Birt et al., 2023).

### Strengths and limitations

As Recovery College dementia courses are a new offering within the Recovery College model there was limited published data to draw on, demonstrating the importance of using a theory driven approach to build the programme theory from diverse sources including the wider literature on Recovery Colleges and discussions with stakeholders. Stakeholders with lived and professional experience of dementia and Recovery College dementia courses have been involved throughout the review. Propositions for how Recovery College dementia courses were thought to work were developed from a co-produced theory of change, and theory development continued for the duration of the review. Discussions with stakeholders with lived experience were fundamental to understanding that if practical and emotional barriers to accessing courses are not addressed then the benefits of the courses will not be realised.

This realist review was completed within a tight timeframe (6 months) to ensure subsequent project timelines and goals were met. Our intention was to provide a sufficiently well-elaborated initial programme theory that would be useful to inform data collection in the next part of the project, which used observations and interviews of Recovery College dementia course co-producers and attendees to collect the primary data needed to further develop and test (confirm, refute, or refine) the initial programme theory (Birt et al., 2023). However, the synthesis continued beyond the six months of the review and included additional searches and analysis. The concepts set out in this initial programme theory were not significantly altered by the additional evidence but understanding of the different components was refined. Steps in the realist review were adapted to meet project timelines while adhering to realist principles; the focus of searches and eligibility criteria were narrowed. We acknowledge this as a limitation and that some relevant data may have been missed. However, primary data collection is a key component of the DiSCOVERY project and will likely address any gaps in the initial programme theory.

## Conclusion

Access to effective post-diagnostic support for people living with dementia is a significant factor in facilitating personal recovery to live with meaning and purpose. For scalability and sustainability of Recovery College dementia courses, it needs to be established how courses can reach people struggling to reconcile their future alongside dementia, and how people living with dementia can be supported emotionally and practically to co-produce and attend courses. A realist evaluation will test and refine the initial programme theory leading to recommendations for future course development.

## Supplementary Material

Supplemental Material

## Data Availability

The search terms and strategies are provided in the Supplementary Material to allow for replication. The data that support the findings of this study are available from the corresponding author, MH, upon reasonable request.
