# Supplementary material for: Operationalising the Recovery College model with people living with dementia: a realist review
Source: Aging Ment Health. 2024 Jun 8;28(8):1078–89. doi: 10.1080/13607863.2024.2356878 (PMC11262432; doi:10.1080/13607863.2024.2356878)
Supplement: Supplemental Material [file CAMH_A_2356878_SM5495.docx]

**Supplementary material 1**

***Co-developing the theory of change map***

Theory-driven evaluation helps us understand why and how programs work by identifying the key components and relationships (Pawson & Tilley, 1997), and mapping the steps from activities to long-term goals using a Theory of Change (ToC) with input from stakeholders is one approach to building understanding (Breuer et al., 2015). This supplementary file provides additional insights and details to accompany the paper titled "Operationalising the Recovery College model with people living with dementia: a realist review", offering a deeper understanding of the methods, ethical considerations, and practical implications of collaboratively developing a ToC as a precursor to a realist review and evaluation.

In the main paper, we outlined the significance of dialogues with people living and working with dementia in the review process to contribute to developing the initial programme theories. This initial participatory process began at research grant application stage and engaged a range of stakeholders including DiSCOVERY study researchers, individuals living with dementia, their family supporters and healthcare staff working in NHS mental health memory services.

***Methods***

*a. Selection of participants:*

Members of the research team contacted a group of healthcare staff (n=3) who were known to be co-producing a Recovery College course about dementia in the NHS Trust leading the proposed research, and invited them to participate in stakeholder engagement activities to support the research grant application. Staff then approached the people with lived experience of dementia and their respective family supporters (dyads n=2, one couple in their 80s and one in their 50s) involved in co-producing the course with a Patient and Public Involvement (PPI) in research recruitment flyer and information sheet and to gain permission for researchers to contact.

*b. Ethical considerations:*

Following initial approach via email, all individuals willingly agreed to share their experiences and insights as part of a PPI in research partnership, and therefore written informed consent was not required. Individuals were assured that insights and responses shared would be paraphrased into relevant sections of the ToC map with no personal identifiable information included.

*c. Practical implications:*

The work to co-develop the ToC map by researchers JW, LB and CH began in January 2020 resulting in the final version (v4 07.09.2020) submitted as part of the research grant application. Due to the onset of the COVID-19 pandemic, planned face-to-face meetings with participants in March 2020 were adapted to using email, telephone, online video-conferencing and printed/posted versions to review, discuss and agree sections of the map. This process was flexible to accommodate the varying cognitive abilities and communication styles of individuals with dementia, and the unique service-level demands on the NHS mental healthcare staff involved at that time.

***Process of co-developing the Theory of Change map***

Development of a scoping survey of UK Recovery College dementia courses (Lowen, Birt and West, 2019) provided background with key papers discussing application of theoretical models of personal recovery and person-centred dementia care in relation to adjusting to a life with a diagnosis of dementia identified. This overview was used by researchers JW, LB and CH to co-develop a first draft of the ToC map. Prior to working with stakeholders a second draft was created by JW using an accessible example provided by danryan.us (<https://djjr-courses.wikidot.com/ppol225-text:charts-and-diagrams-logic-models>). This graphic representation was further co-developed through conversations with researchers LB, CH and RM, and healthcare staff involved in co-producing the local Recovery College dementia course (clinical psychologists n=2). The third version of the map was printed in A3 colour and sent by post to provide individuals living with dementia with a visual aid to support discussions. A Zoom meeting with JW and the two dyads of people with dementia and their family supporters was based around four key questions. The questions were: 1) What is your experience of support since you received the diagnosis?; 2) What has helped you?; 3) What hasn’t helped you?; and 4) What is important to you as a person? This process provided a baseline analysis of the section of the map identifying contexts and issues around problems of post-diagnostic dementia support. Contributions from this meeting specifically influenced the direction of the theory in highlighting the impacts of stigma on the process of adjusting to a diagnosis of dementia and the importance of the unique roles of co-production and peer support in working to support the effects of these negative impacts. To make the theory of change actionable in the context of the Recovery College model, individual staff identified a need for a person-centred approach to co-production, content and delivery of courses about dementia.

***Conclusion***

Integration of lived and learned experiences into the ToC map enriched it with real-world insights, and adapting this co-development approach during the onset of the COVID-19 pandemic accommodated diverse cognitive abilities and communication styles, ensuring the relevance and effectiveness of our research.

***References***

Breuer, E., Lee, L., De Silva, M. et al. Using theory of change to design and evaluate public health interventions: a systematic review. *Implementation Sci* 11, 63 (2015). [https://doi.org/10.1186/
s13012-016-0422-6](https://doi.org/10.1186/s13012-016-0422-6)

Lowen, C., Birt, L., and West, J. Recovery colleges and dementia courses – a scoping survey. *Mental Health and Social Inclusion* 23(4), 166-172 (2019). <https://doi.org/10.1108/MHSI-08-2019-0024>

Pawson, R., & Tilley, N. (1997). Realistic evaluation. Sage Publications.

**Supplementary material 2**

**Supplementary material 3**

Table 1 Search strategy for MEDLINE, EMBASE, PsycINFO, CINAHL 18/01/2022

| Core Search | Supplementary searches |
| --- | --- |
| 1. exp Dementia/  2. (dementia or alzheimer*).ti,ab,kw.  3. 1 or 2  4. (recovery adj2 (college* or approach* or practice* or practise* or ethos or principle* or focus* or framework* or paradigm*)).ti,ab,kw.  5. CHIME.ti,ab,kw.  6. 4 or 5  7. 3 and 6 | 1. recovery college*.ti,ab,kw  2. (experience* or perception* or perspective* or view* or opinion* or satisfaction* or expectation*).ti,ab,kw.  3. (qualitative or mixed method* or evaluation* or interview* or focus group*).ti,ab,kw.  4. exp qualitative research/  5. or/2-4  6. 1 and 5 |

Table 2 Search strategy in other databases 18/01/2022

| Database | Search terms |
| --- | --- |
| Google Scholar (core search) | (dementia OR alzheimers) AND ("recovery college" OR "recovery approach" OR "recovery framework" OR "recovery practice" OR "recovery focus" OR "recovery ethos" OR "recovery paradigm" OR CHIME) |
| Google Scholar (supplementary search) | "recovery college" AND (experience OR perception OR perspective OR view OR opinion OR satisfaction OR expectation OR qualitative OR "Mixed methods" OR evaluation OR interview OR "focus group") |
| NHS Evidence (core search) | 1. (dementia or alzheimers) and ("recovery college" or "recovery approach" or "recovery framework")  2. (dementia or alzheimers) and ("recovery practice" OR "recovery focus" OR CHIME) |
| NHS Evidence (supplementary search) | "recovery college" AND (experience OR perception OR perspective OR view OR opinion OR satisfaction OR expectation OR qualitative OR "Mixed methods" OR evaluation OR interview OR "focus group") |
| NIHR Library | "recovery college"  "recovery approach"  "recovery framework"  "recovery practice"  "recovery focus"  "CHIME" |
